# Tembusu virus induces lipid droplet accumulation to facilitate viral replication in neuronal cells via AMPK-mediated ATGL suppression

**DOI:** 10.1186/s13567-026-01872-1

**Published:** 2026-11-04

**Authors:** Shinuo Fan, Siqi Wang, Chunli Song, Chenyang Song, Guijun Wang, Qing Wang

**Affiliations:** Present Address: College of Veterinary Medicine, Anhui Agricultural University, No.130 Changjiang Road, Hefei, 230036 China

**Keywords:** TMUV, lipid droplets, viral replication, neuropathogenesis, ATGL

## Abstract

Tembusu virus (TMUV) is a neurotropic avian flavivirus that causes viral encephalitis and egg-drop syndrome in ducks, resulting in significant economic losses in duck-producing regions in Asia. Lipid droplets (LDs) are organelles derived from the endoplasmic reticulum that primarily store neutral lipids and are involved in viral replication and pathogenesis. However, little is known about the roles of LDs in TMUV replication and neuropathogenesis. Here, we report that TMUV infection increased triglyceride (TG) levels and LD accumulation in mouse neuroblastoma cells (Neuro-2a). Inhibiting TG synthesis and LD accumulation with specific drugs reduced TMUV replication, whereas oleic acid (OA) supplementation increased LD accumulation and enhanced viral replication, indicating that LDs promote TMUV replication. Furthermore, in TMUV-infected ICR mice or goslings, we assessed LD accumulation in brain tissues and its impact on viral replication and pathology. TMUV infection increased TG levels and LD accumulation in the brain. Inhibiting LD accumulation reduced viral replication and alleviated lesions, whereas enhancing LD accumulation had the opposite effects, indicating that LDs promote TMUV neuropathogenesis. We further explored the mechanism underlying TMUV-induced LD accumulation and revealed that TMUV downregulated the lipolytic enzyme adipocyte triglyceride lipase (ATGL). ATGL overexpression reduced TG production and LD accumulation, thereby inhibiting TMUV replication, whereas ATGL knockdown had the opposite effects. Moreover, TMUV activated AMP-activated protein kinase (AMPK) signalling to suppress ATGL expression, leading to increased TG levels and LD accumulation, thereby increasing viral replication. Collectively, these findings indicate that TMUV infection induced LD accumulation via AMPK-mediated ATGL suppression to promote viral replication, and that LD accumulation contributed to TMUV neuropathogenesis.

**Supplementary Information:**

The online version contains supplementary material available at https://doi.org/10.1186/s13567-026-01872-1.

## Introduction

Tembusu virus (TMUV), first isolated from *Culex* mosquitoes in Malaysia in 1955, is an arthropod-borne RNA virus belonging to the genus *Orthoflavivirus*, family Flaviviridae [1, 2]. The TMUV genome is approximately 11 kb in size and encodes three structural proteins (capsid, premembrane, and envelope proteins) and seven nonstructural proteins (NS1, NS2A, NS2B, NS3, NS4A, NS4B, and NS5) [3]. In 2010, an outbreak of duck TMUV occurred in major duck-raising areas in China [4]. TMUV-induced infections were subsequently reported in other South East Asian countries, including Thailand and Malaysia [5, 6]. TMUV can cause systemic infection in ducks, resulting in severely decreased egg production in laying ducks, retarded growth and neurological diseases in young ducklings, and even death, leading to significant economic losses and restricting the development of the duck farming industry [6, 7].

TMUV can infect not only ducks, but also other avian species, such as chickens, geese, and house sparrows [8, 9]. Moreover, there have recently been reports of natural TMUV infection causing death in bottlenose dolphins [10], and TMUV has also been shown to be pathogenic in mice under artificial experimental conditions, suggesting potential risks of cross-species transmission [11]. Furthermore, a previous study has detected TMUV-positive serum in duck farm workers and TMUV nucleic acid in the oral swabs of some workers [12]. These findings indicated that TMUV is likely to infect people and become a zoonotic pathogen. Like several other flaviviruses that infect humans, such as Japanese encephalitis virus (JEV), dengue virus (DENV), Zika virus (ZIKV), and West Nile virus (WNV), TMUV is also neurotropic and capable of invading the central nervous system, leading to viral encephalitis [13, 14]. Elucidating the neuropathogenesis of TMUV is therefore essential for facilitating the development of effective vaccines and improved control strategies.

Lipids play crucial roles in the lifecycle of neuroinvasive viruses and are related to the neurological damage caused by neuroinvasive virus infection [15, 16]. Lipid droplets (LDs), which are primarily composed of triglycerides (TG) and cholesterol esters, are the main place in which neutral lipids are stored in cells [17]. Diacylglycerol acyltransferase 1 (DGAT1) and DGAT2 convert diacylglycerol (DAG) and fatty acids into TG, which is the main component of LDs in the brain [17, 18]. The perilipin (PLIN) family of proteins, including PLIN1-5, is expressed on the surface of LDs and regulates their stability [19]. The turnover of LDs is a dynamic process, and LDs are continuously generated from the endoplasmic reticulum (ER) and degraded either by adipocyte triglyceride lipase (ATGL), or by lipophagy, a type of selective autophagy [18, 20]. Increasing evidence has shown that various viruses modulate lipid metabolism to favor their replication and contribute to viral pathogenicity [15, 20]. Members of the family Flaviviridae, such as ZIKV, regulate lipid metabolism, leading to significant LD accumulation in human neural cells, which contributes to ZIKV replication and pathogenesis in neural cells [15]. However, the roles of lipid metabolism and LDs in TMUV replication and infection remain unclear. The aim of this study was to investigate the role of LDs in TMUV replication and infection, and to partially elucidate the mechanism by which TMUV exploits lipid metabolism to promote viral replication.

In the present study, we examined the TG production and LD accumulation in TMUV-infected Neuro-2a and mouse brain tissues, and further evaluated their effects on TMUV replication and neuropathogenicity. Next, we investigated the role of ATGL in TMUV-induced TG production and LD accumulation and identified its upstream regulatory signaling molecules. Overall, we elucidated how TMUV regulates ATGL to promote LD accumulation, thereby facilitating viral replication.

## Materials and methods

### Cell culture and viral infection

Baby hamster kidney cells (BHK-21; ATCC: CCL-10) and mouse neuroblastoma cells (Neuro-2a; ATCC: CCL-131) were cultured in Dulbecco’s modified Eagle medium (DMEM; Gibco, USA) supplemented with 10% fetal bovine serum (FBS; Gibco, USA) at 37 ℃ and 5% CO_2_. The TMUV strain AQ-19 (GenBank accession no. MT708901.1) used in this study was preserved in our laboratory and propagated in BHK-21 cells. The virus stock was titrated by determining the 50% tissue culture infective dose (TCID_50_) calculated by the Reed–Muench method in BHK-21 cells. The titer of the virus stock was 10^5.6^ TCID_50_/100 μL. Neuro-2a cells were seeded in 6-well, 24-well, or 96-well plates to 60–70% confluence and then infected with TMUV AQ-19 at a multiplicity of infection (MOI) of 0.5 for all experiments. After 2 h of infection, the cells were washed twice with phosphate-buffered saline (PBS) and then cultured in medium supplemented with 2% FBS for 12, 24, or 36 h.

### BODIPY staining

LD accumulation was analyzed using boron–dipyrromethene (BODIPY) staining following the manufacturer’s protocol. Briefly, Neuro-2a cells were cultured in 24-well plates and infected with TMUV AQ-19 for 12 h, 24 h, and 36 h. The cells were washed with PBS and then fixed with 4% paraformaldehyde for 30 minutes (min) at room temperature. The cells were subsequently permeabilized with 0.1% Triton X-100 solution for 10 min. The LDs were stained with 5 μM BODIPY 493/503 (Glpbio, cat. no. GC42959, USA) and incubated at 37 ℃ in the dark for 30 min. The cells were washed with PBS and stained with 4′,6-diamidino-2-phenylindole (DAPI) (Solarbio, cat. no. ID2250, China) for nuclear staining for 10 min at room temperature. After being washed with PBS, the LDs were observed using a Leica laser scanning confocal fluorescence microscope (STELLARIS5, Leica, Germany). The fluorescence intensity was analyzed using ImageJ software.

### Determination of intracellular triglycerides

Neuro-2a cells were cultured in six-well plates until 60% confluence, and infected with AQ-19 for 12, 24, or 36 h. The cells were added to the cell lysate and allowed to stand for 10 min. After centrifugation, the supernatant was collected for subsequent enzymatic determination. Intracellular TG levels were determined using a TG assay kit (Applygen, cat. no. E1013, China) according to the manufacturer’s instructions. The absorbance was measured at 550 nm using a Multiskan Microplate Photometer (Thermo, USA), and the TG content was calculated according to the standard curve of the absorbance.

### Oil Red O staining

Neuro-2a cells were cultured in 24-well plates and infected with TMUV AQ-19 for 36 h. The infected and mock-infected cells were fixed with 4% formaldehyde for 30 min at room temperature. LDs were stained with Oil Red O solution (Solarbio, cat. no. G1262, China) for 15 min at room temperature. Nuclear recognition was performed with Mayer’s hematoxylin staining solution (Solarbio, cat. no. G1080, China) for 1 min. Images were captured using a light microscope equipped with a 100× objective lens (Olympus, Japan). For brain tissue staining, brain tissues were sectioned and washed three times with PBS. The sections were then stained with Oil Red O solution at room temperature and incubated at 37 °C in the dark. After being washed, the nuclei were counterstained with Mayer’s hematoxylin solution, and the stained sections were observed under a light microscope.

### Cell treatment

To analyze the roles of LDs in TMUV replication, Neuro-2a cells were pretreated with DGAT1 inhibitor A922500 (MCE, cat. no. HY-10038, USA) at 20 μM or DGAT2 inhibitor PF-06424439 (MCE, cat. no. HY-108341, USA) at 140 μM to inhibit the generation of LDs, or oleic acid (OA; Sigma‒Aldrich, cat. no. O1383, USA) at 100 μM to promote the generation of LDs for 2 h before TMUV AQ-19 infection. To analyze the upstream pathways of ATGL, Neuro-2a cells were pretreated with the AMPK inhibitor Compound C (MCE, cat. no. HY-13418A, USA) at 5 μM for 6 h and subsequently infected with TMUV AQ-19. The working concentration and incubation time of each drug were determined on the basis of the cytotoxicity, which was assessed using a CCK-8 solution and through the methods of other references [16, 21, 22].

### Cell viability assay

Cell viability was assessed after treatment with different drugs using a Cell Counting Kit-8 (CCK-8; Beyotime, cat. no. C0038, China). Briefly, Neuro-2a cells were seeded in 96-well plates and cultured overnight to 80–90% confluence. After infection with TMUV AQ-19 for 12 h, 24 h, or 36 h, the cells were treated with fresh medium supplemented with 10% (v/v) CCK-8 solution, with wells without cells serving as the background control. The plates were incubated in the dark at 37 °C for 2 h, after which the absorbance was measured at 450 nm using a Multiskan Microplate Photometer (Thermo, USA). Three repeated samples were used in all the experiments, and the results were verified by conducting three independent experiments.

### Plasmid construction and transfection

On the basis of the published sequence of the ATGL gene (GenBank accession no. OP393009.1), primers were designed using SnapGene software to amplify the full-length gene. The primers used in this study are listed in Table 1. Total RNA was extracted from Neuro-2a cells using the RNAeasy™ Animal RNA Isolation Kit with a Spin Column (Beyotime, cat. no. R0026, China), complementary DNA (cDNA) was synthesized using HiScript II Q RT SuperMix for qPCR (+ gDNA wiper) (Vazyme, cat. no. R223-01, China), and the ATGL gene was amplified using cDNA as a template. The amplified fragment was subsequently ligated into the pCMV14-HA vector using *Sal* I and *Kpn* I restriction sites to construct the eukaryotic expression plasmid HA-ATGL, and the constructed plasmid was confirmed by sequencing. Neuro-2a cells were cultured to 60–70% confluence in six-well plates, and were transfected with the HA-ATGL plasmid using Lipofectamine 3000 (Invitrogen, cat. no. L3000008, USA), with an empty pCMV14-HA plasmid used as a control. The overexpression of ATGL in transfected cells was confirmed at 24 h and 36 h post-transfection by western blotting.

**Table 1:** **Primers used in this study**

Gene	Sequence (5′–3′)	Product size
NS5	AGGAAGTGGAGCAATCAGGAA	180 bp
	TAACAAGTGGCAGAGCAAAGG	
GAPDH (mice)	GAAGGTGGTGAAGCAGGCATCT	116 bp
	CGGCATCGAAGGTGGAAGAGTG	
GAPDH (goslings)	CCGAGAGGCAAGATGGTGAAA	90 bp
	GACTTTGCCAGACAGGATGG	
SREBP1	GGATGCGGCTGTTGTCTACCATAAG	87 bp
	GCCAGGTTAGAAGCAGCAAGATGT	
SREBF2	CGAAGTGGAAGAACTGAGATGC	187 bp
	GGCTGAATGACCGCTGTACT	
PPARα	AGAATCCACGAAGCCTACCTGAAGA	196 bp
	GGAAGAATCGGACCTCTGCCTCT	
PPARβ	GTAGAAGCCATCCAGGACACCATTC	179 bp
	AGCAAGGTCTCACTCTCCGTCTT	
PPARγ	TCGCCAAGGTGCTCCAGAAGAT	103 bp
	TGAAGGCTCATGTCTGTCTCTGTCT	
PLIN2 (mice)	TCCACTCCACTGTCCACCTGAT	122 bp
	GCCGATGCTTCTCTTCCACTCC	
PLIN2 (goslings)	GCTGCCTATAAGTCGCGGG	128 bp
	TTCAGTCTTACACAGCCGCA	
PLIN3	AGCAGCGACAGGAGCAGAACT	90 bp
	GCTTGCCGAGGGAGTGTTCATAG	
ATGL	CCTTCGCAATCTCTACCGCCTCT	199 bp
	CTCCACCACAGCAGCTTCCTCT	
HSL	ACAGAGACACCAGCCAACGGATA	101 bp
	TTGCGGTTAGAAGCCACATAGCG	
DGAT1	GCCCAAGGTAGAAGAGGACG	163 bp
	CAGACGATGGCACCTCAGAT	
DGAT2	AGCACCCGACCCAGAAAGACAT	125 bp
	TCAGTTCACCTCCAGCACCTCAG	
HA-ATGL	ACGCGTCGACGCCACCATGTTCCCGAGGGAGACCA	1482 bp
	GGGGTACCGCAAGGCGGGAGGCCAG	
AMPK	CGTCGCCTACCACCTCATCATAGA	147 bp
	TCGGCAACCAAGAACGGTACTCT	

### RNA interference

Three small interfering RNAs (siRNAs) targeting ATGL and a nontargeting siRNA (siRNA-NC) were designed and synthesized by Tsingke (Beijing, China). Three different siRNAs targeting ATGL used were as follows: siRNA-1-ATGL, 5′-GUGAAGCAGGUGCCAACAUUA-3′; siRNA-2-ATGL, 5′-GCUGAAGAAUACCAUCACA-3′; and siRNA-3-ATGL, 5′-CUACAGAGAUGGACUUCGA-3′. Neuro-2a cells were seeded in six-well plates and cultured to 80% confluence. After washing three times with PBS, Neuro-2a cells were transfected with the siRNA using Lipofectamine RNAiMAX (Invitrogen, cat. no. 13778075, USA) according to the manufacturer’s instructions. Cells transfected with siRNA-NC were used as a negative control. Cells transfected with siRNA were identified by western blotting and quantitative reverse transcription polymerase chain reaction (qRT‒PCR) at 24 h and 36 h post-transfection.

### qRT‒PCR

Total RNA was extracted from cells using the RNAeasy™ Animal RNA Isolation Kit with a Spin Column (Beyotime, cat. no. R0026, China), and complementary DNA (cDNA) was synthesized using the HiScript II Q RT SuperMix for qPCR (+ gDNA wiper) (Vazyme, cat. no. R223-01, China). The amplification reaction was performed using the ChamQ Universal SYBR qPCR Master Mix (Vazyme, cat. no. Q711-02, China) and a 7500 Real-Time PCR System (Applied Biosystems). The *NS5* gene expression levels were detected using absolute qRT‒PCR to assess TMUV replication. The *GAPDH* gene was used as an internal control, and the expression levels of genes related to lipid droplet metabolism results were calculated by the CT method (2^−ΔΔCT^). The primers used in this study are listed in Table 1. All qRT‒PCR experiments were performed in triplicate, and each experiment was repeated three independent times.

### Western blot analysis

Neuro-2a cells were collected and lysed using radioimmunoprecipitation assay (RIPA) buffer (Solarbio, cat. no. R0010, China) supplemented with protease and phosphatase inhibitors for 10 min on ice. The protein concentration was measured using a bicinchoninic acid (BCA) Protein Quantification Kit (Abbkine, cat. no. KTD3001, China) according to the manufacturer’s instructions. The samples were mixed with 5× sodium dodecyl sulfate (SDS)‒polyacrylamide gel electrophoresis (PAGE) loading buffer and boiled at 100 °C for 10 min, after which an equal amount of protein sample was added for SDS‒PAGE. The protein was transferred to a polyvinylidene fluoride (PVDF) membrane (Millipore, USA) using the wet transfer method. The membrane was blocked with 5% nonfat milk in Tris-buffered saline and Tween 20 (TBST) for 2 h, followed by incubation with the specified primary antibody overnight at 4 °C. The membrane was washed with TBST to remove unbound primary antibody and then incubated with the corresponding secondary antibody at 37 °C for 1 h. After the membrane was washed again with TBST, the enhanced chemiluminescence (ECL) solution (Proteintech, cat. no. PK10001, China) was added for exposure, and the band intensity was quantified using ImageJ. The primary antibodies used in this study were as follows: TMUV NS1 rabbit polyclonal antibody (prepared in our laboratory), HA tag rabbit polyclonal antibody (Proteintech, cat. no. 51064-2-AP, China), PLIN2 rabbit monoclonal antibody (Abcam, cat. no. ab108323, UK), ATGL rabbit polyclonal antibody (Proteintech, cat. no. 55190-1-AP, China), AMPK alpha (D5A2) rabbit monoclonal antibody (CST, cat. no. 5831, USA), phospho-AMPK alpha rabbit monoclonal antibody (CST, cat. no. 2535, USA), and β-actin mouse monoclonal antibody (Servicebio, cat. no. GB15001-100, China). The secondary antibody used for western blotting was horseradish peroxidase (HRP)-conjugated goat anti-mouse immunoglobulin G (IgG) (1:8000; Solarbio, cat. no. SE131, China) or HRP-conjugated goat anti-rabbit IgG (1:8000; Solarbio, cat. no. SE134, China).

### Animal experiments

The effect of LDs on AQ-19-induced brain injury was evaluated on the basis of an ICR mouse model infected with TMUV previously established in the laboratory [23]. Thirty-six female ICR mice aged 3 weeks were purchased from the Laboratory Animal Center of Anhui Medical University. Female mice were selected based on our previously established TMUV infection model to maintain consistency with previous studies [11, 23]. Furthermore, using mice of the same sex may help minimize potential variability associated with sex-related physiological differences. The animals were placed in an independently ventilated cage, and pathogen-free food and water were provided. After adaptive prefeeding for 3 days, the mice were randomly divided into four groups (*n* = 9 per group): the AQ-19 group, OA + AQ-19 group, A922500 + AQ-19 group, and control group. Intraperitoneal injection (i.p.) of A922500 (2.5 mg/kg of body weight) and oral administration of OA (400 mg/kg of body weight) were performed 2 h before infection, followed by administration every 24 h. Mice in the AQ-19 infection group and the control group were treated with normal saline. In addition to the control group, each group of mice was inoculated intracranially with 30 μL of AQ-19 virus stock solution (TCID_50_/0.1 mL = 10^5.6^), and the control group was inoculated with 30 μL of DMEM. The injection site was located at the midpoint of the line connecting the anterior margins of the left and right external auditory canals along the sagittal midline. The needle was inserted vertically through the skull to a depth of approximately 5 mm, and the inoculum was then delivered intracranially. The mice in the AQ-19 infection group were euthanized at 2, 4, and 6 days after infection (dpi), and the remaining mice were sacrificed at 6 dpi. Brain tissues were collected for western blotting, qRT‒PCR, Oil Red O staining, and immunohistochemistry (IHC) or hematoxylin and eosin (H&E) staining.

In addition, we used goslings, the natural host of TMUV, to conduct the above experiments. Briefly, 24 healthy 1-day-old goslings were obtained from a healthy flock from Anhui Province and acclimated for 3 days before being randomly assigned to four groups (*n* = 6 per group): the AQ-19 group, OA + AQ-19 group, A922500 + AQ-19 group, and control group. The drug administration regimen, including the dose, route, and timing, was identical to that used in the mouse infection experiments. Goslings were challenged intramuscularly with 0.5 mL of AQ-19 virus stock solution, while control animals received an equal volume of DMEM. All goslings were euthanized at 5 dpi, and brain tissues were collected for subsequent analyses.

### Statistical analysis

All experiments were conducted independently three times. The values are expressed as the mean ± standard deviation (SD). Statistical analysis was performed using GraphPad Prism 8 software (La Jolla, CA, USA). The significance of differences between groups was assessed using Student’s *t* test and one-way analysis of variance (ANOVA). The confidence interval was set at 95%. A *p* value less than 0.05 was considered to indicate statistical significance.

## Results

### TMUV infection promotes TG synthesis and induces LD accumulation in Neuro-2a cells

**Figure 1:**
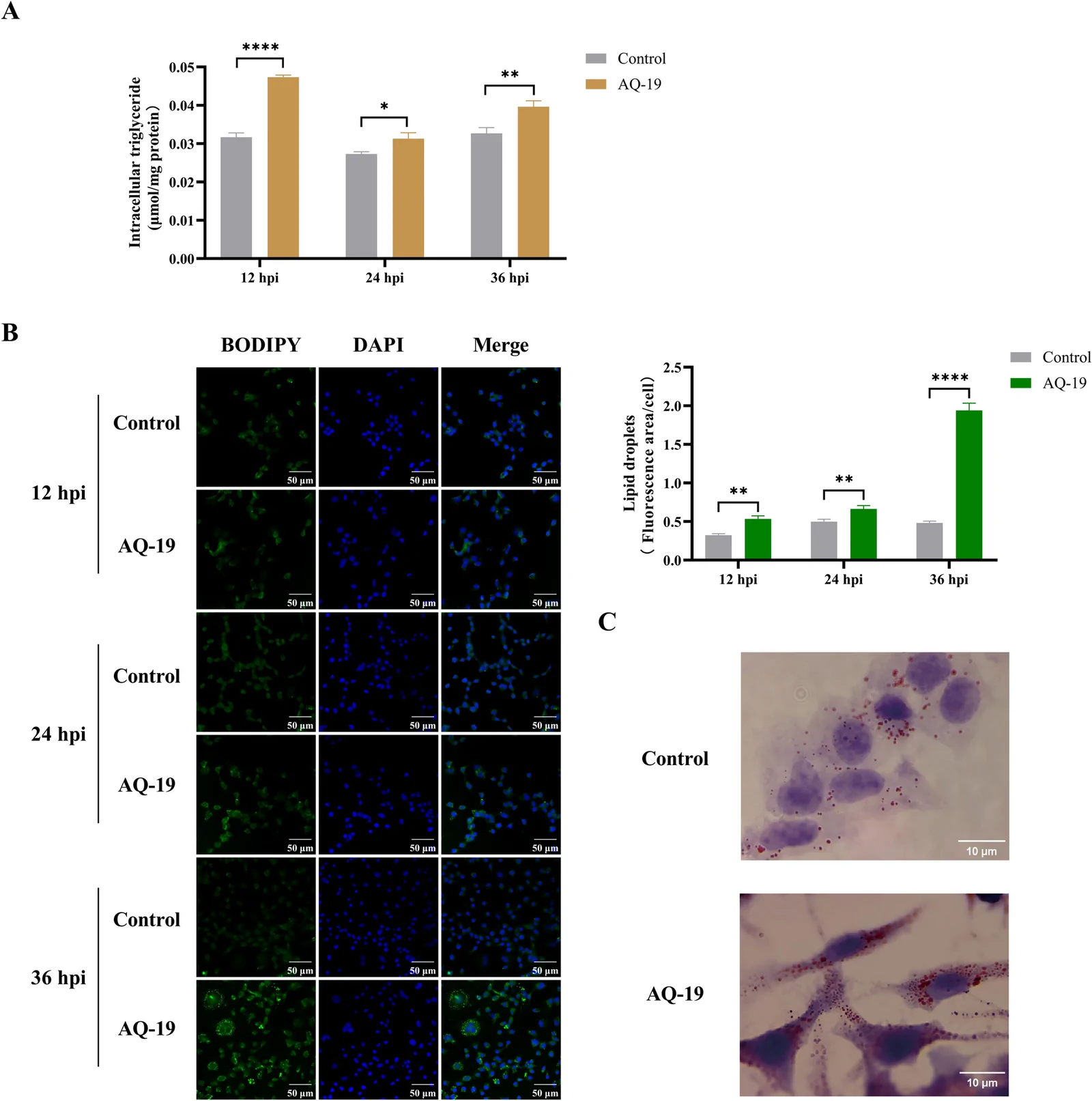
**TMUV infection increases TG levels in Neuro-2a cells and induces LD accumulation**. **A** Neuro-2a cells were uninfected or infected with TMUV, and the intracellular TG levels were measured at 12, 24 and 36 hpi. **B** Neuro-2a cells were left uninfected or infected with TMUV. At 12, 24, and 36 hpi, the cell samples were collected and stained with BODIPY 493/503 (green) to label the LDs and DAPI (blue) to stain the nuclei. LD accumulation was observed using confocal microscopy. Scale bar: 50 μm. LD content was quantified using ImageJ software (*n* = 3). **C** LDs in uninfected Neuro-2a cells and Neuro-2a cells infected with TMUV for 36 h were detected by Oil Red O staining. Scale bar: 10 μm. Differences were evaluated using Student’s* t* test. *, *p* < 0.05; **, *p* < 0.01; ****, *p* < 0.0001.

Neuro-2a cells were infected with the TMUV AQ-19 strain at an MOI of 0.5, and the intracellular TG levels were measured at 12, 24, and 36 hpi. The results showed that TMUV infection significantly increased TG production in Neuro-2a cells (Figure 1A). Since TGs are the major components of LDs, we next investigated whether TMUV infection induces LD accumulation in Neuro-2a cells. Confocal microscopy analysis revealed that the number of LDs in Neuro-2a cells gradually increased over time following infection with the AQ-19 strain (Figure 1B). In addition, Oil Red O staining performed at 36 hpi further confirmed that TMUV infection markedly increased LD accumulation in Neuro-2a cells (Figure 1C).

### TG synthesis and LD accumulation facilitate TMUV replication

**Figure 2:**
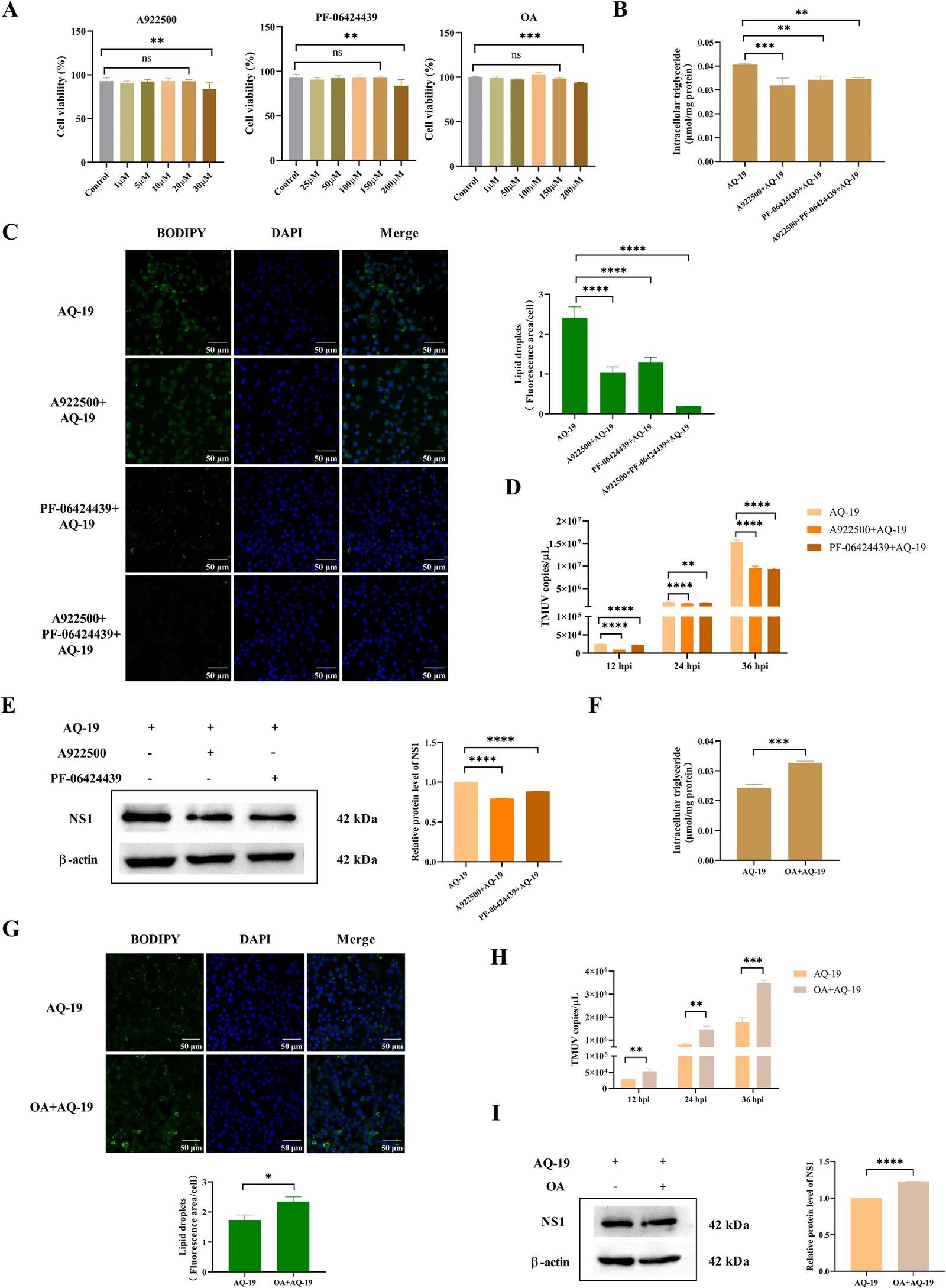
**TG production and LD accumulation promote TMUV replication in Neuro-2a cells. A** The cell viability of Neuro-2a cells was assessed via CCK-8 assay following treatment with the DGAT1 inhibitor A922500, the DGAT2 inhibitor PF-06424439, and the LDs inducer OA. **B, F** Neuro-2a cells were pretreated with DMSO (0.1%), A922500 (20 μM), PF-06424439 (140 μM), or OA (100 μM) for 2 h, followed by infection with TMUV AQ-19. At 36 hpi, cell samples were collected and intracellular TG levels were measured. **C, G** Neuro-2a cells were treated with the indicated drugs and infected with TMUV for 36 h (as described in **B**). Cells were collected and stained with BODIPY 493/503 (green) and DAPI (blue). LD accumulation was observed using confocal microscopy. Scale bar: 50 μm. The LD content was quantified using ImageJ software (*n* = 3). **D, H** Neuro-2a cells were treated with the indicated drugs, then infected with TMUV and collected at the indicated time points. qRT‒PCR was performed to evaluate the number of TMUV copies. **E, I** Neuro-2a cells were treated with the indicated drugs and infected with TMUV for 36 h, after which western blotting was performed to evaluate the levels of the TMUV NS1 protein. Band intensities were calculated and analyzed using ImageJ software. Differences were analyzed using one-way ANOVA (**A**–**E**) and Student’s *t* test (**F**–**I**). *ns* not significant; *, *p* < 0.05; **, *p* < 0.01; ***, *p* < 0.001; ****, *p* < 0.0001.

DGATs, including DGAT1 and DGAT2, are key enzymes involved in TG synthesis and LD formation [24]. We first determined the noncytotoxic concentrations of the DGAT1 inhibitor A922500, the DGAT2 inhibitor PF-06424439, and the LD inducer OA. On the basis of the cell viability results, Neuro-2a cells were pretreated with A922500 (20 μM), PF-06424439 (140 μM), or OA (100 μM) prior to infection with the TMUV AQ-19 (Figure 2A). The results showed that pretreatment with either A922500 or PF-06424439 significantly reduced the intracellular TG levels in the AQ-19-infected cells (Figure 2B). Consistently, confocal microscopy analysis demonstrated that inhibition of TG synthesis markedly attenuated TMUV-induced LD accumulation in Neuro-2a cells (Figure 2C).

To further investigate the role of TG synthesis and LD accumulation in TMUV replication, viral replication was subsequently evaluated. The results revealed that inhibition of TG synthesis and LD accumulation significantly reduced viral copy numbers and the expression of the viral protein NS1 (Figure 2D, E), indicating suppressed viral replication. To further validate the roles of TG and LDs in TMUV replication, Neuro-2a cells were pretreated with OA prior to TMUV infection. OA supplementation increased TMUV-induced TG synthesis and LD accumulation, which was accompanied by increased viral replication (Figure 2F–I). Collectively, these results demonstrate that TG synthesis and LD accumulation promote TMUV replication.

### TMUV infection induces TG production and LD accumulation in the mouse brain and enhances viral neuropathogenesis

**Figure 3:**
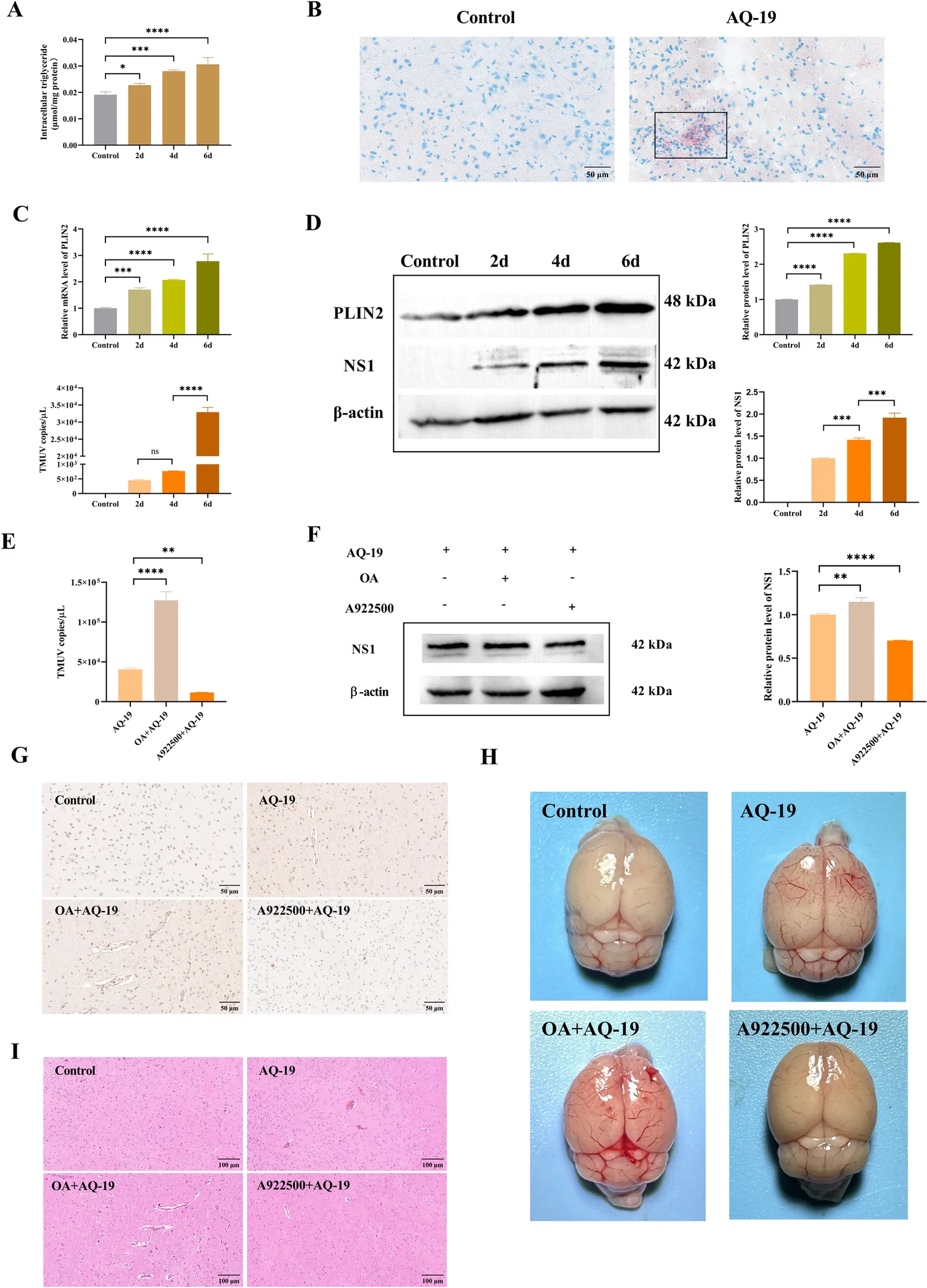
**TMUV infection induces LD accumulation in the mouse brain, promoting viral replication and neurovirulence. A** TG levels in mouse brain tissues were measured at 2, 4, and 6 days after TMUV infection. **B** LDs in the brain tissues of mice were detected by Oil Red O staining 6 days after infection with TMUV. The black box shows the accumulation of LDs in the brain tissue. Scale bar: 50 μm. **C** The mRNA levels of PLIN2 and the viral copy numbers in the brain tissues of mice infected with TMUV for 2, 4, and 6 days were determined via qRT‒PCR. **D** The protein expression levels of PLIN2 and viral NS1 in the brain tissues of mice infected with TMUV for 2, 4, and 6 days were determined via western blotting. **E, F** Brain tissues from mice infected with TMUV for 6 days were collected, and viral copy numbers and NS1 protein expression levels were determined via qRT‒PCR and western blotting, respectively. The protein band intensities in **D** and **F** were quantified using ImageJ software. **G** Immunohistochemical (IHC) staining was used to observe the distribution of TMUV antigens (brown) in mouse brain tissues. Scale bar: 50 μm. **H, I** Gross pathological examination and H&E staining were performed to observe histopathological changes in mouse brain tissues. Scale bar: 100 μm. The results were analyzed using one-way ANOVA. *, *p* < 0.05; **, *p* < 0.01; ***, *p* < 0.001; ****, *p* < 0.0001.

On the basis of our in vitro findings that LD accumulation promotes TMUV replication in Neuro-2a cells, an ICR mouse infection model was used to further validate these observations in vivo and to evaluate the impact of LD accumulation on TMUV-induced neuropathogenesis. TG levels in mouse brain tissues gradually increased at 2, 4, and 6 dpi (Figure 3A). Consistently, Oil Red O staining of brain tissues at 6 dpi revealed marked LD accumulation in TMUV-infected mice (Figure 3B). To further characterize LD accumulation in the brain, the transcriptional and protein expression levels of the LDs-associated protein PLIN2 were examined. In parallel, viral copy numbers and NS1 protein expression in brain tissues were also detected. The results showed that TMUV infection significantly upregulated both the mRNA and protein levels of PLIN2 in mouse brain tissues, with the peak expression detected at 6 dpi, which was consistent with the increased viral replication in the brain (Figure 3C, D). Collectively, these findings further confirm that TMUV infection induces LD accumulation in the mouse brain, which is consistent with the in vitro results.

To evaluate the role of LD accumulation in TMUV replication, qRT‒PCR, western blotting, and IHC analyses were performed. The qRT‒PCR and western blotting results showed that, compared with the AQ-19 group, both viral copy numbers and NS1 protein expression levels in brain tissues were significantly greater in the OA+AQ-19 group, whereas they were markedly lower in the A922500+AQ-19 group (Figure 3E, F). Consistently, IHC staining revealed the strongest positive signals in the OA+AQ-19 group, followed by the AQ-19 group, while the weakest signals were observed in the A922500+AQ-19 group, indicating that A922500 treatment effectively inhibited TMUV replication in mouse brain tissues (Figure 3G). Gross pathological examination of the brain tissues revealed that the lesions in the OA+AQ-19 group were more severe than those in the AQ-19 group, whereas pathological damage was markedly alleviated in the A922500+AQ-19 group (Figure 3H). Consistent with these findings, H&E staining revealed that the OA+AQ-19 group displayed the most severe histopathological changes, characterized by inflammatory cell infiltration and perivascular cuffing, whereas compared with the AQ-19 group, the A922500+AQ-19 group presented substantially milder lesions (Figure 3I). Collectively, these results indicate that TMUV-induced LD accumulation promotes viral replication in mouse brain tissues and exacerbates viral neuropathogenesis.

Additionally, TMUV-induced LD accumulation was confirmed in the brain tissues of AQ-19-infected goslings. As shown in Additional file 1A and B, AQ-19 infection significantly increased TG levels and induced marked LD accumulation in the brains of infected goslings. In addition, AQ-19 infection significantly upregulated both the mRNA and protein levels of PLIN2 in the brain tissues of infected goslings (Additional file 1C, D). Furthermore, we found that viral replication in the brain was significantly higher in the OA + AQ-19 group than in the AQ-19 group, whereas it was significantly reduced in the A922500 + AQ-19 group (Additional file 1E–G). Gross pathological examination and H&E staining further demonstrated that the OA + AQ-19 group exhibited the most severe brain lesions, whereas the A922500 + AQ-19 group showed markedly attenuated pathological changes (Additional file 1H, I). These findings further demonstrate that TMUV infection induces LD accumulation in the brain of its natural avian host, thereby promoting viral replication and aggravating TMUV-induced neuropathogenesis.

### TMUV infection downregulates ATGL expression in Neuro-2a cells

**Figure 4:**
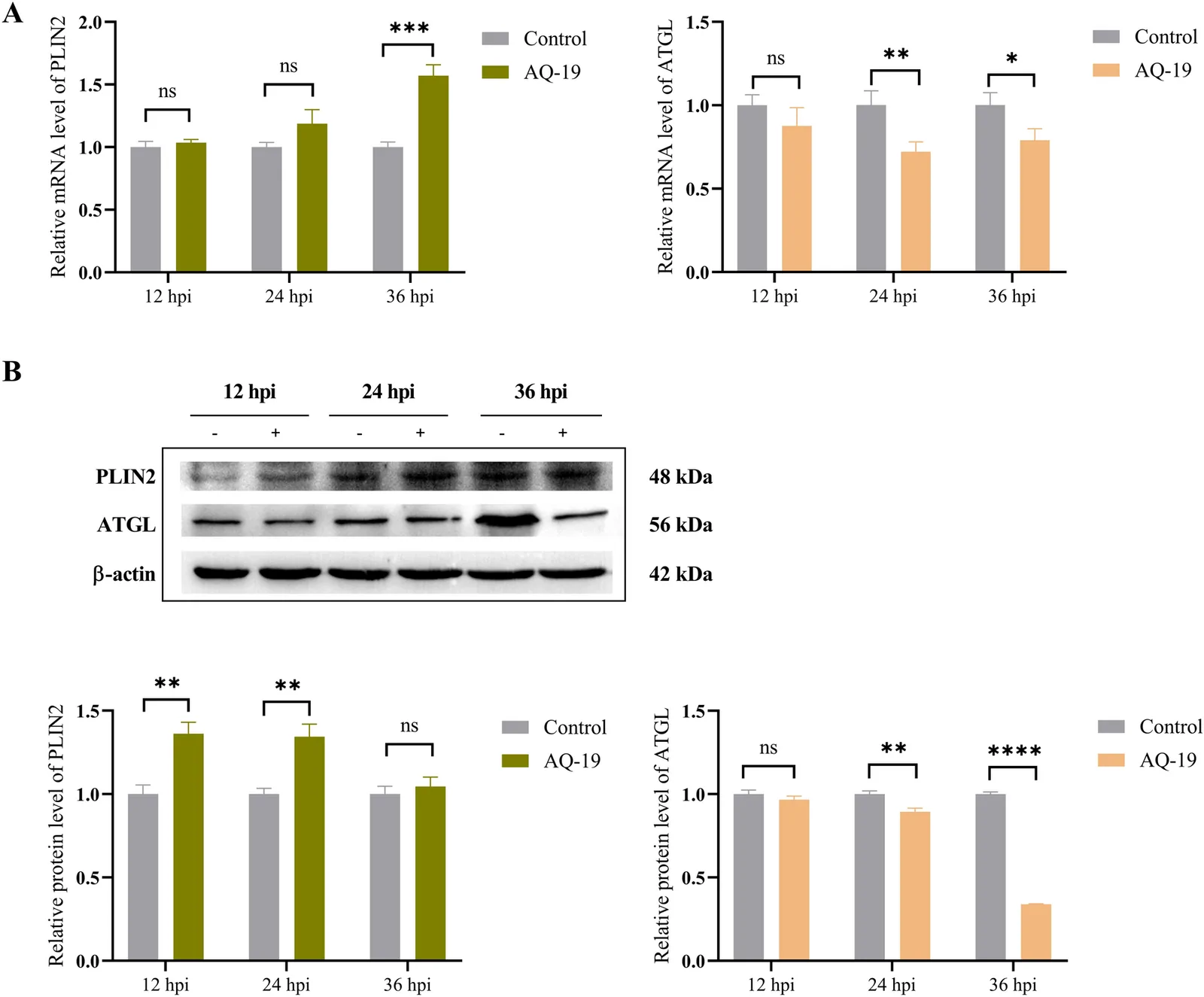
**TMUV infection downregulates ATGL expression.** Neuro-2a cells were uninfected or infected with TMUV. At 12, 24, and 36 hpi, cell samples were collected. qRT‒PCR (**A**) and western blotting (**B**) were carried out to determine the expression levels of PLIN2 and ATGL. Protein band intensities were quantified using ImageJ software. Significant differences were determined using Student’s *t* test. *ns* not significant; *, *p* < 0.05; **, *p* < 0.01; ***, *p* < 0.001; ****, *p* < 0.0001.

ATGL, encoded by the *Pnpla2* gene, is a rate-limiting enzyme in lipolysis that initiates TG hydrolysis by cleaving the first ester bond of TG, thereby providing substrates for subsequent lipid degradation. ATGL is localized mainly on the surface of cytoplasmic LDs and other intracellular membranes. Upon recruitment to LD membranes, ATGL initiates the hydrolysis of TG [25, 26]. To investigate the effect of TMUV infection on ATGL expression, the transcriptional and protein levels of PLIN2 and ATGL were detected in Neuro-2a cells at 12, 24, and 36 hpi. TMUV infection significantly upregulated both the mRNA and protein levels of PLIN2, whereas ATGL expression was markedly downregulated compared with that in the control group (Figure 4A, B). These findings suggest that TMUV infection induces LD accumulation in Neuro-2a cells while suppressing ATGL-mediated lipolysis. In addition, the transcription levels of several LD metabolism-related genes were analyzed. The results showed that the transcription level of *Plin3*, which is associated with newly formed LDs, was upregulated at 36 hpi. The transcription level of *Srebp1*, a key regulator of LDs homeostasis, was significantly upregulated at 12 and 36 hpi. In contrast, no significant changes were observed in the expression of *Srebp2*, a major regulator of cholesterol biosynthesis. Moreover, the transcription level of *Hsl*, another lipolytic enzyme, was downregulated only at 12 hpi (Additional file 2). Collectively, these results indicate that TMUV-induced LD accumulation is primarily associated with increased PLIN2 expression and the suppression of ATGL-mediated TG hydrolysis, whereas the cholesterol synthesis pathway appears to play a limited role in this process.

### Overexpression of ATGL reduces LD accumulation and inhibits TMUV replication

**Figure 5:**
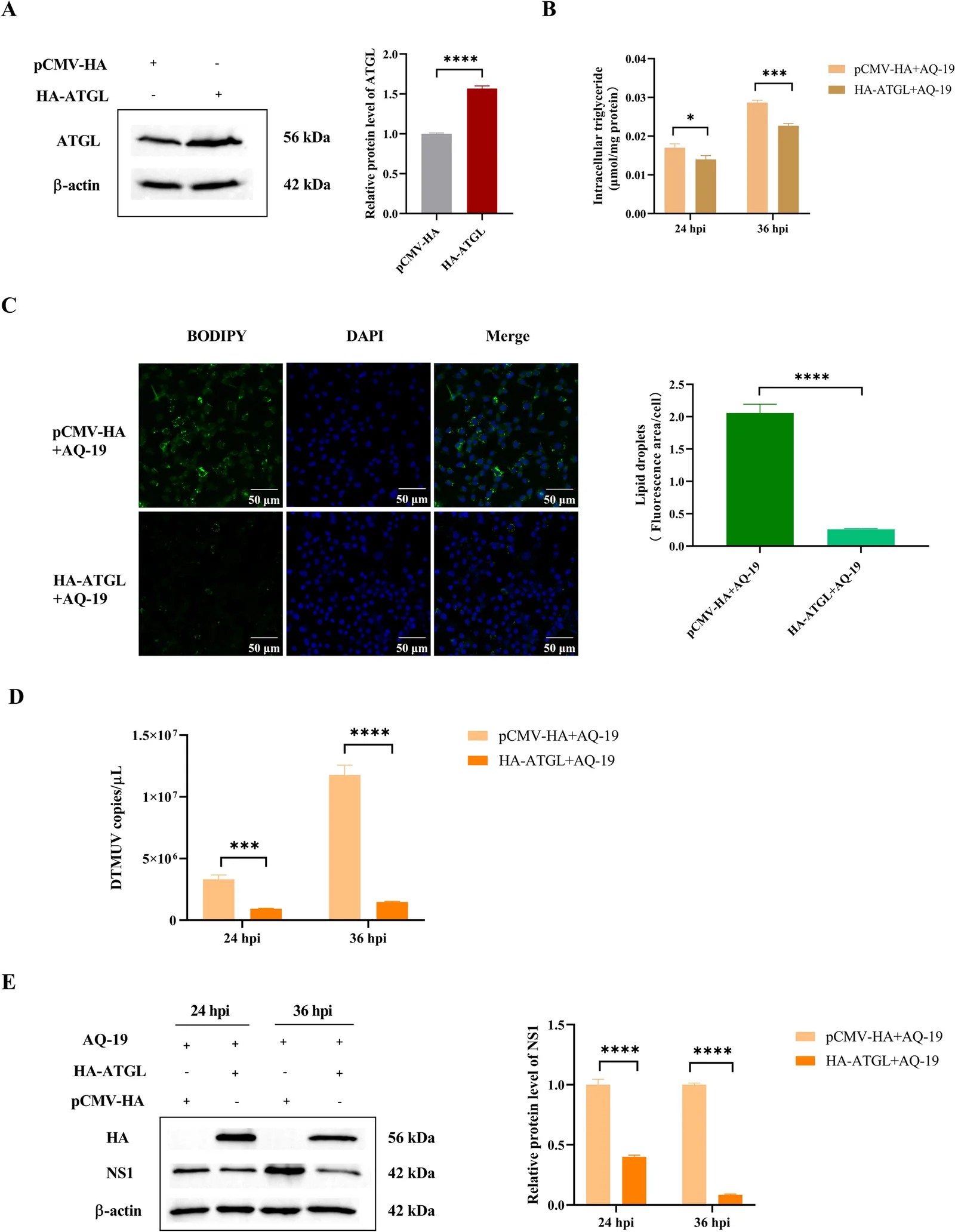
**ATGL overexpression reduces LD accumulation and inhibits TMUV replication. A** Neuro-2a cells were transfected with pCMV14-HA or HA-ATGL for 36 h. The expression level of ATGL was evaluated via western blotting. **B, D, E** At 6 h post-transfection, Neuro-2a cells were infected with TMUV AQ-19, and cell samples were collected at 24 hpi and 36 hpi. Intracellular TG levels were measured (**B**). Viral copy numbers and NS1 protein expression levels were determined via qRT‒PCR (**D**) and western blotting (**E**). Protein band intensities were quantified using ImageJ software. **C** Neuro-2a cells transfected with pCMV14-HA or HA-ATGL for 6 h were infected with TMUV AQ-19 for 36 h. The cells were stained with BODIPY 493/503 (green) and DAPI (blue). LD accumulation was observed using confocal microscopy. Scale bar: 50 μm. The LD content was quantified using ImageJ software (*n* = 3). Significant differences were determined using Student’s *t* test. *ns* not significant; *, *p* < 0.05; ***, *p* < 0.001; ****, *p* < 0.0001.

To explore the role of ATGL in regulating TG synthesis and LD accumulation during TMUV infection, Neuro-2a cells were transfected with either pCMV-HA or HA-ATGL plasmids. Western blot analysis performed 36 h after transfection confirmed the successful overexpression of ATGL protein (Figure 5A). Subsequently, Neuro-2a cells were transfected with pCMV-HA or HA-ATGL plasmids for 6 h and then infected with the TMUV AQ-19 strain. Intracellular TG levels were measured at 24 and 36 hpi. Compared with those in the pCMV-HA vector control group, the TG levels in the HA-ATGL-transfected cells were significantly lower (Figure 5B). To further evaluate LD accumulation, the cells were collected at 36 hpi and stained with BODIPY 493/503, followed by confocal microscopy analysis. As shown in Figure 5C, compared with that in the pCMV-HA control group, LD accumulation in HA-ATGL-transfected Neuro-2a cells was markedly reduced. These findings indicate that ATGL overexpression significantly suppresses TMUV-induced TG production and LD accumulation in Neuro-2a cells.

TMUV replication was subsequently assessed via qRT‒PCR and western blotting analysis. The results of the qRT‒PCR analysis showed that viral copy numbers in HA-ATGL-transfected Neuro-2a cells were significantly lower than those in the pCMV-HA vector control group at both 24 and 36 hpi (Figure 5D). Consistently, western blot analysis performed at 36 hpi revealed that the viral NS1 protein level in HA-ATGL-transfected Neuro-2a cells was markedly lower than that in the pCMV-HA control group (Figure 5E). Collectively, these findings indicate that ATGL overexpression effectively inhibits TMUV replication in Neuro-2a cells.

### Knockdown of ATGL increases LD accumulation and promotes TMUV replication

**Figure 6:**
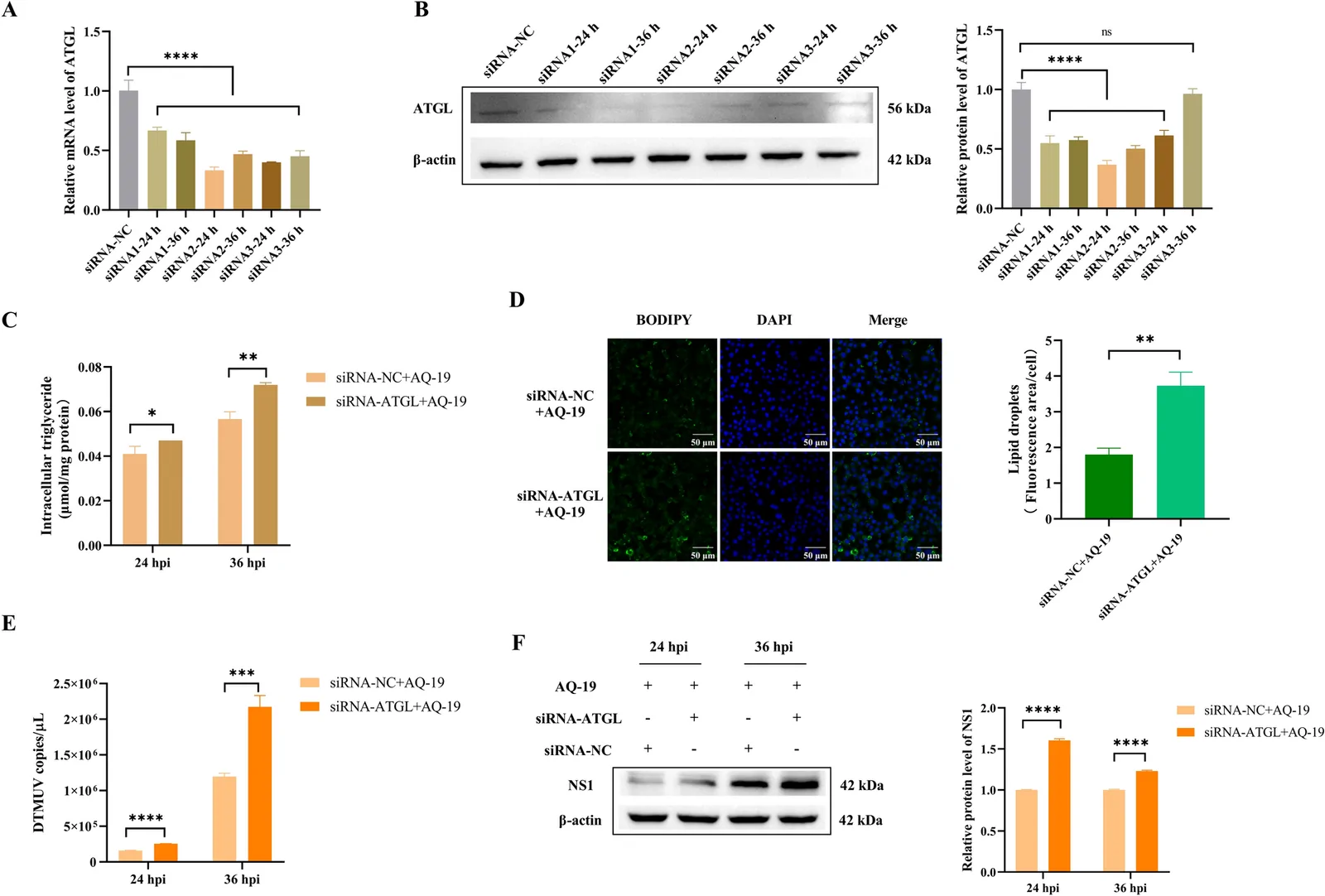
**ATGL silencing increases LD accumulation and promotes TMUV replication. A, B** Neuro-2a cells were transfected with siRNAs targeting ATGL or negative control siRNA (siRNA-NC) for 24 h and 36 h. The mRNA and expression levels of ATGL were evaluated via qRT‒PCR (**A**) and western blotting (**B**), respectively. **C, E, F** At 6 h post-transfection, Neuro-2a cells were infected with TMUV AQ-19, and cell samples were collected at 24 hpi and 36 hpi. Intracellular TG levels were measured (**C**). Viral copy numbers and NS1 protein expression levels were determined via qRT‒PCR (**E**) and western blotting (**F**). Protein band intensities were quantified using ImageJ software. **D** Neuro-2a cells were transfected with siRNA-2 or siRNA-NC for 6 h and then were infected with TMUV AQ-19 for 36 h. The cells were stained with BODIPY 493/503 (green) and DAPI (blue). LD accumulation was observed using confocal microscopy. Scale bar: 50 μm. LD content was quantified using ImageJ software (*n* = 3). Significant differences were determined using one-way ANOVA (**A**, **B**) and Student’s *t* test (**C**–**F**). *ns* not significant; *, *p* < 0.05; **, *p* < 0.01; ***, *p* < 0.001; ****, *p* < 0.0001.

Three siRNAs targeting *ATGL* were designed and transfected into Neuro-2a cells. The knockdown efficiency of these siRNAs was evaluated via qRT‒PCR and western blot analysis. Compared with those in the siRNA-NC group, both the mRNA and protein levels of ATGL in the cells transfected with siRNA-2 were markedly lower (Figure 6A, B), indicating that siRNA-2 effectively silenced ATGL expression in Neuro-2a cells. To further investigate the role of ATGL in regulating TG synthesis and LD accumulation during TMUV infection, Neuro-2a cells were transfected with siRNA-2 for 6 h and then infected with the TMUV AQ-19 strain, and intracellular TG levels were measured at 24 and 36 hpi. The results showed that the TG levels in the siRNA-2 group were significantly greater than those in the siRNA-NC group (Figure 6C). To assess LD accumulation, the cells were collected at 36 hpi, stained with BODIPY 493/503, and analyzed by confocal microscopy. LD accumulation was markedly greater in the siRNA-2 group than in the siRNA-NC group (Figure 6D). These findings indicate that TMUV promotes TG synthesis and LD accumulation by suppressing ATGL expression.

Furthermore, the effect of ATGL knockdown on TMUV replication was assessed via qRT‒PCR and western blot analysis. Compared with those in the siRNA-NC group, the viral copy numbers in the siRNA-2 group significantly increased at both 24 and 36 hpi (Figure 6E). Western blot analysis performed at 24 and 36 hpi demonstrated that the expression level of the viral NS1 protein was markedly greater in the siRNA-2 group than in the siRNA-NC group (Figure 6F). Collectively, these findings indicate that ATGL knockdown promotes TMUV replication in Neuro-2a cells.

In addition, to validate the role of ATGL using a pharmacological approach, Neuro-2a cells were pretreated with the ATGL inhibitor Atglistatin for 2 h prior to TMUV infection. Based on the results of the cell viability assay, a concentration of 20 μM Atglistatin was selected for subsequent experiments (Additional file 3A). Consistent with the effects of ATGL knockdown, Atglistatin treatment significantly increased intracellular TG levels and promoted LD accumulation in TMUV-infected cells (Additional file 3B, C). In addition, pharmacological inhibition of ATGL markedly enhanced TMUV replication, as evidenced by increased viral copy numbers and viral NS1 protein expression (Additional file 3D, E). These findings further confirm that inhibition of ATGL promotes TMUV-induced LD accumulation and facilitates viral replication.

### AMPK–ATGL signaling plays an important role in TMUV-induced LD accumulation

**Figure 7:**
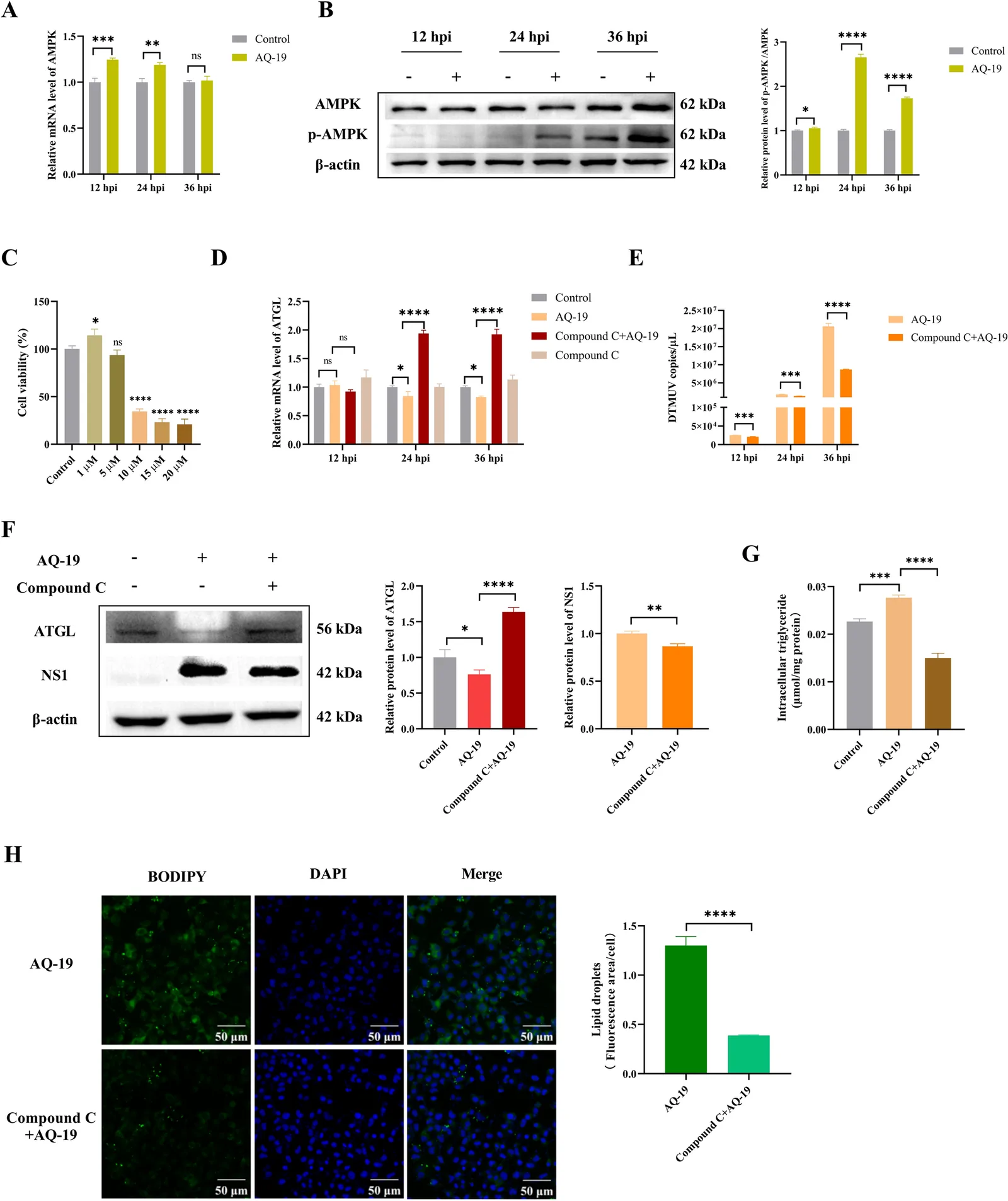
**TMUV infection induces LD accumulation in Neuro-2a cells via the AMPK–ATGL axis. A, B** Neuro-2a cells were uninfected or infected with TMUV AQ-19. At 12, 24, and 36 hpi, cell samples were collected. The mRNA level of AMPK was determined via qRT‒PCR (**A**). The expression level of p-AMPK was evaluated via western blotting (**B**). **C** The cell viability of Neuro-2a cells was assessed via CCK-8 assay following treatment with the AMPK inhibitor Compound C. **D** Uninfected Neuro-2a cells and Neuro-2a cells were infected with TMUV for 12, 24, and 36 h after treatment with or without Compound C. The mRNA level of ATGL was determined via qRT‒PCR. **E** Neuro-2a cells were infected with TMUV for 12, 24, and 36 h after treatment with or without Compound C. Viral copy numbers were determined via qRT‒PCR. **F, G** Uninfected Neuro-2a cells and Neuro-2a cells were infected with TMUV for 36 h after treatment with or without Compound C. The expression levels of ATGL and viral NS1 protein were evaluated via western blotting (**F**). Intracellular TG levels were measured (**G**). **H** Neuro-2a cells were infected with TMUV for 36 h after treatment with or without Compound C. The cells were stained with BODIPY 493/503 (green) and DAPI (blue). LD accumulation was observed using confocal microscopy. Scale bar: 50 μm. The LD content was quantified using ImageJ software (*n* = 3). Significant differences were determined using Student’s *t* test (**A**, **B**, **E** and **H**) and one-way ANOVA (**C**, **D**, **F** and **G**). *ns* not significant; *, *p* < 0.05; **, *p* < 0.01; ***, *p* < 0.001; ****, *p* < 0.0001.

AMP-activated protein kinase (AMPK) is a central regulator of cellular energy homeostasis and is involved in the regulation of protein, glucose, and lipid metabolism, as well as autophagy and mitochondrial function. In adipose tissue, AMPK regulates lipid metabolism by modulating key triglyceride lipases, including ATGL [27]. In this study, the transcriptional and protein levels of AMPK, as well as its phosphorylation (p-AMPK), were analyzed in Neuro-2a cells infected with the TMUV AQ-19 strain at 12, 24, and 36 hpi. The results of the qRT‒PCR analysis showed that AMPK mRNA levels were significantly higher in the AQ-19-infected group than in the control group at 12 and 24 hpi, whereas no significant difference was detected at 36 hpi (Figure 7A). In contrast, western blot analysis demonstrated that, compared with those in the control group, both total AMPK protein levels and p-AMPK levels significantly increased at 12, 24, and 36 hpi (Figure 7B).

To further investigate the role of AMPK in regulating ATGL expression and TMUV replication, the cytotoxicity of the AMPK inhibitor Compound C was first evaluated, and 5 μM was selected as the working concentration for subsequent experiments (Figure 7C). Neuro-2a cells were pretreated with Compound C for 6 h and then infected with the AQ-19 strain, and ATGL expression and viral replication were subsequently evaluated via qRT‒PCR and western blot analysis. Compared with those in the AQ-19 group, ATGL mRNA levels in the Compound C + AQ-19 group did not significantly differ at 12 hpi but did significantly increase at 24 and 36 hpi (Figure 7D). In parallel, viral copy numbers were significantly lower in the Compound C + AQ-19 group than in the AQ-19 group at 12, 24, and 36 hpi (Figure 7E). Consistently, western blot analysis at 36 hpi showed that ATGL protein levels were markedly upregulated, whereas the viral NS1 protein levels were significantly decreased in the Compound C + AQ-19 group (Figure 7F). Collectively, these results indicate that TMUV downregulates ATGL expression in an AMPK-dependent manner, and that AMPK activation promotes viral replication in Neuro-2a cells.

To further explore the role of AMPK in regulating TG synthesis and LD accumulation during TMUV infection, Neuro-2a cells were pretreated with Compound C and subsequently infected with the TMUV AQ-19 strain. At 36 hpi, the intracellular TG levels were measured. TG synthesis was significantly lower in the Compound C + AQ-19 group than in the AQ-19 group (Figure 7G). Consistently, BODIPY 493/503 staining followed by confocal microscopy demonstrated a marked decrease in LD accumulation in the Compound C + AQ-19 group (Figure 7H). Collectively, these results demonstrate that TMUV infection induces LD accumulation by downregulating ATGL expression in an AMPK-dependent manner.

## Discussion

As an emerging mosquito-borne flavivirus, TMUV has caused serious threats to the poultry industry because of its wide transmission and expanding host range, and it also poses potential risks to mammalian and human health [28, 29]. Therefore, it is necessary to further investigate the infection mechanisms of TMUV to provide a basis for developing novel strategies to control its infection and spread. In this study, we revealed that LDs promote TMUV replication in neuronal cells and that LD accumulation enhances the neuropathogenesis of TMUV infection. We further investigated the molecular mechanism underlying TMUV-induced LD accumulation and identified the AMPK–ATGL axis as a key regulator that promotes LD accumulation and facilitates viral replication.

LDs are dynamic organelles that primarily store lipids, especially TG, and play crucial roles in maintaining cellular lipid and energy homeostasis. They also act as hubs that regulate diverse metabolic processes and exhibit a wide range of biological functions [30]. Owing to their lack of autonomous metabolic capacity, viruses must rely on host cells to complete their life cycle. Recent studies have shown that various viruses can exploit LDs and LD-related components to facilitate their replication and pathogenesis [31]. For instance, rabies virus infection enhanced LD formation, which consequently facilitated viral budding and eventually promoted viral replication [32], and porcine reproductive and respiratory syndrome virus infection increased intracellular TG levels within the host cells, contributing to LD accumulation and viral replication [20]. Our study demonstrated that TMUV infection led to a significant increase in TG levels and LD accumulation in Neuro-2a cells. Consistent with our findings, members of the *Flavivirus* genus, such as Zika virus (ZIKV) and hepatitis C virus (HCV), have been reported to manipulate host lipid metabolism and induce LD accumulation during infection [33, 34]. DGATs, including DGAT1 and DGAT2, are key rate-limiting enzymes that catalyze the final step of TG biosynthesis [35]. In this study, inhibition of DGATs to block LD accumulation reduced TMUV replication in Neuro-2a cells. In contrast, OA-induced increases in TG levels and LD formation promoted the replication of TMUV.

ICR mice or goslings were subsequently infected with TMUV, and TMUV infection induced increased TG levels and LD accumulation in the mouse or gosling brain, which is consistent with the in vitro results. To further assess the impact of LDs on disease severity, mice or goslings were treated with the DGAT1 inhibitor A922500 or the LD inducer OA. The viral load in the brain tissues of the mice or goslings in the A922500 + AQ-19 group was lower than that in the AQ-19 and OA + AQ-19 groups, and the severity of the brain lesions was less severe in the A922500 + AQ-19 group than in the other two groups. Similarly, ZIKV infection alters lipid metabolism in human neural cells, promotes LD formation, and provides a favorable environment for viral replication. Treatment with A922500 reduced LD accumulation and ZIKV replication in a mouse infection model and increased survival by 75% [15]. Moreover, IAV infection disrupts lipid metabolism in mouse lung tissue, leading to LD accumulation, in which the lipid synthesis regulator SREBP2 plays a key role. Inhibiting SREBP2 precursor maturation with fatostatin reduces IAV-induced LD accumulation, improves survival in infected mice, and alleviates lung injury [24]. These results indicate that, like other viruses, TMUV infection promotes LD accumulation, thereby increasing viral replication and pathogenesis. Although further studies are needed, targeting LD formation may represent a promising therapeutic strategy against TMUV infection.

To further explore the mechanism of TMUV-induced LD accumulation, we analyzed the transcription levels of several lipid metabolism-related genes and, notably, found that TMUV infection downregulated ATGL expression. ATGL is a rate-limiting enzyme in adipocyte lipolysis and is localized on LDs and ER-related membranes in the cytoplasm [36]. Upon association with the LD membrane, ATGL initiates TG hydrolysis [25, 26]. It has been reported that viruses can regulate lipid metabolism by modulating ATGL. The HCV nucleocapsid core protein reduces lipolysis by engaging ATGL and induces steatosis in cultured cells and mouse livers [37]. Porcine reproductive and respiratory syndrome virus Nsp4 induces ATGL degradation, leading to increased intracellular TG levels and thereby promoting PRRSV replication and LD accumulation [20]. In our study, ATGL overexpression reduced both intracellular TG levels and LD accumulation, and inhibited TMUV replication, whereas ATGL knockdown increased TG levels, promoted LD accumulation, and further enhanced TMUV replication. These results collectively indicate that TMUV downregulates ATGL expression to inhibit TG hydrolysis, thereby promoting LD accumulation and facilitating viral replication.

AMP-activated protein kinase (AMPK) is a heterotrimeric protein complex composed of three subunits. As a key energy sensor, it plays a crucial role in regulating various intracellular metabolic processes [38]. Previous studies have shown that AMPK is an upstream regulator of ATGL and is involved in the regulation of lipid metabolism [39, 40]. Our previous study demonstrated that TMUV infection activates AMPK, induces incomplete autophagy, and promotes viral replication in Neuro-2a cells [11]. Therefore, we further examined whether AMPK regulates ATGL to influence LD formation. We found that treatment with the AMPK inhibitor Compound C restored the TMUV-induced downregulation of ATGL, suppressed TG synthesis, and reduced the accumulation of intracellular LDs. Moreover, Compound C treatment also inhibited TMUV replication in Neuro-2a cells. These results indicate that TMUV promotes its own replication by inducing LD accumulation via the AMPK–ATGL axis. Consistent with our findings, infection with the bovine viral diarrhea virus (BVDV) also promotes viral replication via the AMPK–ATGL pathway. However, BVDV infection regulates the AMPK–PNPLA2/ATGL signaling to induce lipophagy, thereby mobilizing energy to support its replication, in which the nonstructural protein NS5A plays an important role [27]. Determining which TMUV protein is responsible for regulating the AMPK–ATGL axis to induce LD accumulation requires further investigation.

In conclusion, our findings demonstrated that TMUV induced increased TG levels and LD accumulation, thereby enhancing viral replication and neuropathogenicity. We also revealed that the increase in TG content and LD accumulation is mediated by AMPK-regulated ATGL expression. This study helps to elucidate the role of lipid metabolism in TMUV infection, and the findings provide novel potential targets for the development of anti-TMUV drugs.

## Supplementary Information

**Additional file 1. TMUV infection induces LD accumulation in the gosling brain, promoting viral replication and neurovirulence.** (A) TG levels in gosling brain tissues were measured at 5 days after TMUV infection. (B) LDs in the brain tissues of goslings were detected by Oil Red O staining 5 days after infection with TMUV. The black box shows the accumulation of LDs in the brain tissue. Scale bar: 50 μm. (C) The mRNA levels of PLIN2 and the viral copy numbers in the brain tissues of goslings infected with TMUV for 5 days were determined via qRT‒PCR. (D) The protein expression levels of PLIN2 and viral NS1 in the brain tissues of goslings infected with TMUV for 5 days were determined via western blotting. (E, F) Brain tissues from goslings infected with TMUV for 5 days were collected, and viral copy numbers and NS1 protein expression levels were determined via qRT‒PCR and western blotting, respectively. The protein band intensities in D and F were quantified using ImageJ software. (G) IHC staining was used to observe the distribution of TMUV antigens (brown) in gosling brain tissues. Scale bar: 50 μm. (H, I) Gross pathological examination and H&E staining were performed to observe histopathological changes in gosling brain tissues. Scale bar: 100 μm. The results were analyzed using Student’s* t *test (A, C, D) and one-way ANOVA (E, F). **, *p *< 0.01; ***, *p *< 0.001; ****, *p *< 0.0001.

**Additional file 2. Effects of TMUV on the expression of LDs-related genes in Neuro-2a cells.** Neuro-2a cells were seeded in six-well plates and cultured at 37 °C in a 5% CO₂ incubator. When the cells reached 70% confluence, they were infected with the TMUV AQ-19 strain at an MOI of 0.5. Cell samples were collected 12, 24, and 36 hpi, and qRT‒PCR was performed to detect the expression levels of the LD-related genes: *Pln3*,* Hsl*,* Srebp1*, and *Srebp2*. Significant differences were determined using Student’s *t*-test. *ns* not significant; *, *p* < 0.05; ****, *p* < 0.0001.

**Additional file 3. Inhibition of ATGL increases LD accumulation and promotes TMUV replication.** (A) The cell viability of Neuro-2a cells was assessed via CCK-8 assay following treatment with the ATGL inhibitor Atglistatin. (B, D, E) Neuro-2a cells were pretreated with DMSO (0.1%) or Atglistatin (20 μM) for 2 h, followed by infection with TMUV AQ-19. At 24 and 36 hpi, cell samples were collected and intracellular TG levels were measured (B). Viral copy numbers and NS1 protein expression levels were determined via qRT‒PCR (D) and western blotting (E). Protein band intensities were quantified using ImageJ software. (C) After treatment with DMSO (0.1%) or Atglistatin (20 μM), Neuro-2a cells were infected with TMUV for 36 h. Cells were collected and stained with BODIPY 493/503 (green) and DAPI (blue). LD accumulation was observed using confocal microscopy. Scale bar: 50 μm. The LD content was quantified using ImageJ software (*n* = 3). Significant differences were determined using one-way ANOVA (A) and Student’s* t *test (B-E). *ns* not significant; *, *p* < 0.05; ****, *p* < 0.0001.

## Data Availability

All data supporting the findings of this study are available within the paper and its Supplementary Information.
